# Discovery and Mechanism of Action of a Novel Antimicrobial Peptide from an Earthworm

**DOI:** 10.1128/spectrum.03206-22

**Published:** 2023-01-05

**Authors:** Yizhao Wu, Songge Deng, Xiuhong Wang, Michelle Thunders, Jiangping Qiu, Yinsheng Li

**Affiliations:** a School of Agriculture and Biology, Shanghai Jiao Tong University, Shanghai, China; b Shanghai Yangtze River Delta Eco-environmental Change and Management Observation and Research Station, Ministry of Science and Technology, Shanghai, China; c Department of Pathology and Molecular Medicine, University of Otago, Wellington, New Zealand; University of North Carolina at Chapel Hill

**Keywords:** antibacterial activity, antimicrobial peptide, bacterial membrane, earthworm, molecular simulation

## Abstract

The robust innate immune system of the earthworm provides a potential source of natural antimicrobial peptides (AMPs). However, the cost and high rediscovery rate of direct separation and purification limits their discovery. Genome sequencing of numerous earthworm species facilitates the discovery of new antimicrobial peptides. Through predicting potential antimicrobial peptides in the open reading frames of the Eisenia andrei genome and sequence optimization, a novel antimicrobial peptide, named EWAMP-R (RIWWSGGWRRWRW), was identified. EWAMP-R demonstrated good activity against various bacteria, including drug-resistant strains. The antibacterial mechanisms of EWAMP-R were explored through molecular simulation and wet-laboratory experiments. These experiments demonstrated that the bacterial membrane may be one of the targets of EWAMP-R but that there may be different interactions with Gram-negative and Gram-positive bacterial membranes. EWAMP-R can disrupt bacterial membrane integrity; however, at low concentrations, it appears that EWAMP-R may get through the membrane of Escherichia coli instead of damaging it directly, implying the existence of a secondary response. Gene expression studies identified that in E. coli, only the apoptosis-like cell death (ALD) pathway was activated, while in Staphylococcus aureus, the MazEF pathway was also upregulated, limiting the influence of the ALD pathway. The different antimicrobial actions against Gram-positive and -negative bacteria can provide important information on the structure-activity relationship of AMPs and facilitate AMP design with higher specificity. This study identified a new source of antibacterial agents that has the potential to address the increasingly serious issue of antibiotic resistance.

**IMPORTANCE** Drug-resistant bacteria are a great threat to public health and drive the search for new antibacterial agents. The living environment of earthworms necessitates a strong immune system, and therefore, they are potentially a rich resource of novel antibiotics. A novel AMP, EWAMP-R, with high antibacterial activity was found through *in silico* analysis of the Eisenia andrei genome. Molecular analysis investigating the interactions between EWAMP-R and the cell membrane demonstrated the importance of tryptophan and arginine residues to EWAMP-R activity. Additionally, the different secondary responses found between E. coli and S. aureus were in accordance with a common phenomenon where some antibacterial agents only target specific species of bacteria. These results provided useful molecular information to support further AMP research and design. Our study expands the sources of antimicrobial peptides and also helps to explain the adaptability of earthworms to their environment.

## INTRODUCTION

Earthworms are an important component of terrestrial ecosystems and widely distributed all over the world, demonstrating their potent adaptability to different soil environments (1). Moreover, the existence of earthworms can significantly change the microbial population in the soil, implying strong interactions between earthworms and microorganisms and the robust innate immune system of earthworms (2). In their innate immune system, earthworms can also provide antimicrobial peptides (AMPs), which may result from their potent adaptability to different soil environments (1, 3). AMPs are widely distributed in natural organisms from prokaryotes to humans. After absorption on the cell surface, many AMPs can form membrane channel architectures with diverse structures, leading to the leakage of inner contents and resulting in cell death (4). Although extracellular peptidases, cell wall lipases and flopases, endogenous transporters, and other strategies in bacteria can all contribute to AMP resistance, drug-AMP combinations can also counter those mechanisms (5). Additionally, some AMPs can also have nonmembranolytic activities, such as combining with bacterial membrane components and various intracellular targets (6, 7).

Polymyxin resistance can be attained via horizontal gene transfer. Once referred to as one of the last antibiotic groups, resistance to it has now spread to many countries across at least four continents (8, 9). With the time to launch a new antibiotic estimated at around 15 years (10), antibiotic resistance in bacteria is a matter of global concern, and its horizontal transfer in the ecosystem aggravates the risk to public health. Years of research have confirmed that antimicrobial peptides can be suitable as alternative antibacterial drugs (11, 12) and have the potential to be used against antibiotic-resistant bacteria (13).

Despite AMPs having potent antibacterial activity, the screening approach in the wet laboratory is time costly, has a high rediscovery rate, and would neglect the AMPs in biological materials that are difficult to obtain (6, 14, 15). Therefore, statistical-learning and optimization-based approaches were introduced to predict AMPs from DNA and amino acid sequences (4, 16). On this basis, many databases have been developed to discover natural products from both the microbiome and the genome. Among six general prediction tools based on amino acid sequences, CAMP_R3_ (RF [random forests]) is reported to have the best performance (17). With CAMP_R3_, AMP prediction, antimicrobial-region analysis, and rational design are integrated (18). Through these functions, it is possible to find and optimize AMPs from the vast amount of genomic and proteomic data in publicly available sequence databases.

With the deepening of genome research, the observation of the adaptability of organisms to different environments, and the expansion of knowledge on the molecular structure-activity relationships of proteins, a large number of novel therapeutic drugs can be discovered. In this study, we selected earthworms for novel AMP discovery because of the long-term coevolution of earthworm with soil bacteria and also for their strong regenerative capacity (19). As an important part of their innate defense mechanism, AMPs play a considerable role in protecting earthworms from microbial infection and soil contamination. Unsurprisingly, some earthworm AMPs have been identified from different species; for example, lumbricin-1 from Lumbricus rubellus (Hoffmeister, 1843), PP-1 from Metaphire tshiliensis (Michaelsen, 1928) (formerly Pheretima tschiliensis), lumbricin-PG from Metaphire guillelmi (Michaelsen, 1985) (formerly Pheretima guillelmi), and Lumbr and LuRP from Eisenia andrei (Bouché, 1972) (3). However, the process of extraction, separation, purification, and identification of AMPs from the earthworm body can be time consuming and may omit some key active ingredients. The discovery of homologues of a recognized AMP can also be limited by the acquisition of different species of earthworms.

The objective of this study was to discover new AMPs from the earthworm genome by following three steps: (i) predict potential antibacterial peptides by analyzing open reading frames (ORFs) of the earthworm genome, (ii) modify the structure of these peptides to obtain the optimum antibacterial effect, and (iii) analyze the mechanisms of antibacterial action.

## RESULTS AND DISCUSION

### EWAMP-R is an efficient AMP against drug-resistant bacteria.

On the basis of 2.03 Gb of open reading frames (ORFs) found, 21 peptides were selected for analysis of their MICs (Table S1 in the supplemental material), and 5 of them exhibited antibacterial activity (MIC ≤ 1,024 μg/mL) (Table S2). The lowest MICs (Escherichia coli MIC = 128 μg/mL and Staphylococcus aureus MIC = 128 μg/mL) were shown for EWAMP.15 (MLRKVGVIGHWKIWWSGGWKRWRWR).

Eight peptides with different partial amino acid residues of EWAMP.15 were synthesized to identify the active region (Table S3). In observations of the antibacterial activity of the first half of the sequence of EWAMP.15, the MIC of residues 2 to 14 of EWAMP.15 (EWAMP.15 2–14) showed a substantial decrease compared to the MIC of EWAMP.15 2–15, with only one tryptophan residue difference in the C terminus, indicating that tryptophan residues may play an important role in the antibacterial activity. According to previous studies (20), some tryptophan- and arginine-rich AMPs have strong effects against bacteria. Hence, residues 11 to 25, which include six tryptophan and three arginine residues, should be considered to confirm the active region. Among the AMPs that originated from the sequence of residues 11 to 25, EWAMP.15 12–24 exhibited good antibacterial activity (E. coli MIC = 16 μg/mL and S. aureus MIC = 16 μg/mL).

**FIG 1:**
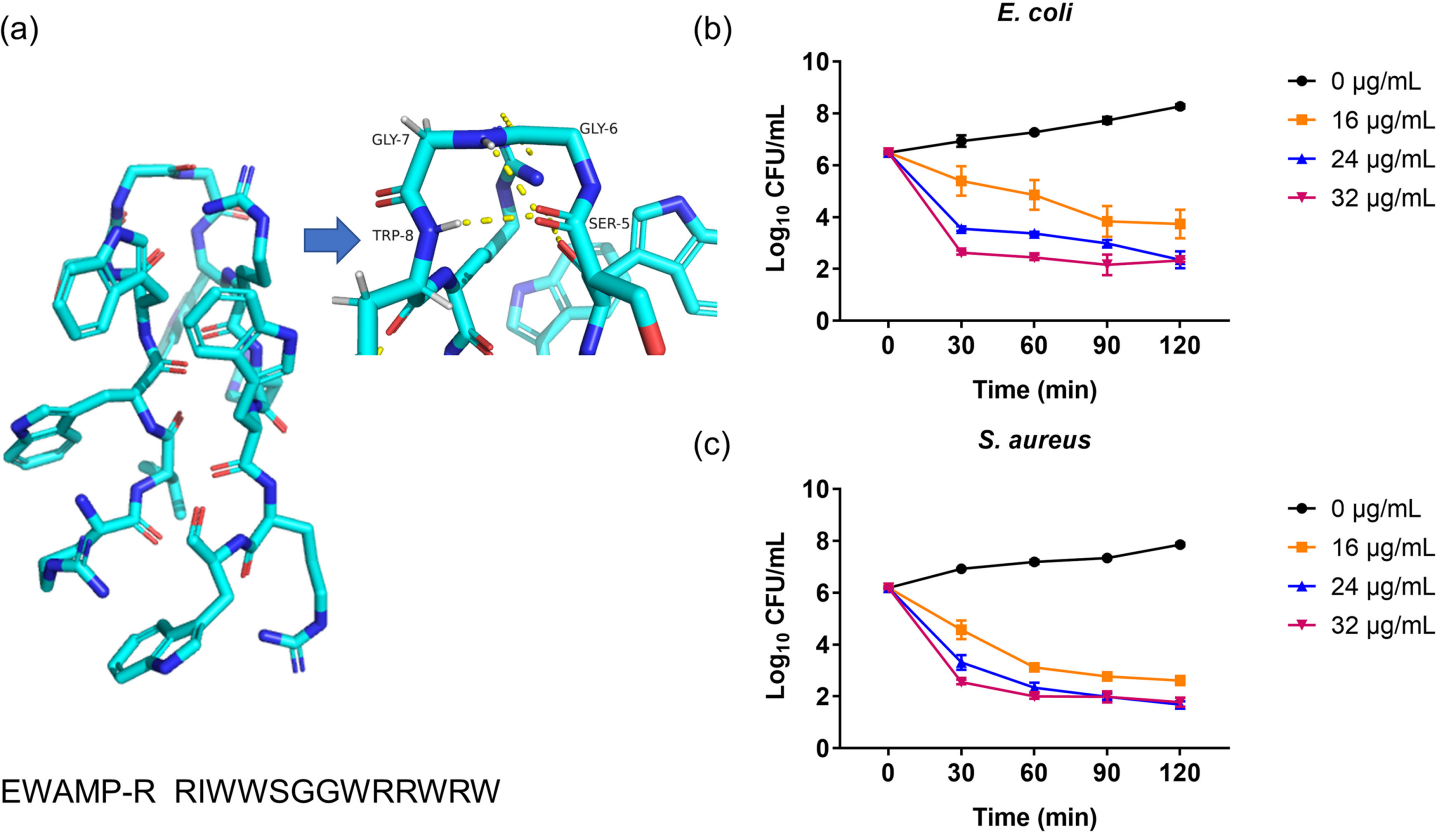
Antimicrobial activity of EWAMP-R. (a) Structure of EWAMP-R predicted by C-I-TASSER, observed by PyMOL. The arrow points to a partial enlarged view, and the yellow lines represent hydrogen bonds. (b) Antimicrobial kinetics of EWAMP-R against E. coli strain ATCC 25922 (*n* = 3). (c) Antimicrobial kinetics of EWAMP-R against S. aureus strain ATCC 29213 (*n* = 3). Keys give the concentrations of EWAMP-R in culture medium.

The structure-function relationship of EWAMP.15 12–24 was further analyzed by alanine scanning and substitutions of other amino acid residues (Table S4, Fig. S1). Significant declines in bacteriostatic activity were observed with the replacement of Trp residues, Lys9, and Arg10, supporting the importance of tryptophan and cationic amino acids. Considering the different interactive effects between Lys, Arg, and Trp residues, more derivatives were synthesized. A better AMP, EWAMP.15 12–24 01 09R (RIWWSGGWRRWRW; designated EWAMP-R the 1st and 9th lysine residues were replaced by arginine residues) (Fig. 1a), was found, with an E. coli MIC of 8 μg/mL and an S. aureus MIC of 16 μg/mL (Table S5). From these data, we concluded that EWAMP-R was likely to have a potency in terms of antibacterial efficiency against E. coli similar to those of antibiotics like ampicillin, cefuroxime, and chloramphenicol (E. coli MIC = 2 to 8 μg/mL) (21), and it could inhibit the growth of bacteria within 30 min *in vitro* (Fig. 1b and 1c). EWAMP-R also showed effective activity against other bacteria, such as Serratia marcescens, Cronobacter sakazakii, and Shigella dysenteriae, and could inhibit drug-resistant bacteria, including extended-spectrum β-lactamase (ESBL) E. coli, carbapenem-resistant E. coli, and methicillin-resistant S. aureus (MRSA) (Table 1). In addition, the antibacterial activity of EWAMP-R showed comparative stability under pH and temperature changes, especially in E. coli (Fig. S2a and b). Early *in vitro* hemolysis and cytotoxicity were not found with a concentration of 256 μg/mL (Fig. S2c and d).

**TABLE 1:** MICs of EWAMP-R against standard strains and clinical isolates of bacteria

Strain	Species	Drug resistance	Origin	MIC [μg/mL (nmol/mL)]
ATCC 25922	Escherichia coli		ATCC	8 (4.24)
ATCC 29213	Staphylococcus aureus		ATCC	16 (8.47)
ATCC 29212	Enterococcus faecalis		ATCC	64 (33.90)
CGMCC 1.4256	Serratia marcescens		CGMCC	16 (8.47)
CGMCC 1.10754	Salmonella enterica subsp. *enterica*		CGMCC	64 (33.90)
CGMCC 1.2022	Enterobacter cloacae		CGMCC	128 (67.79)
CGMCC 1.6197	Yersinia mollaretii		CGMCC	64 (33.90)
CGMCC 1.6765	Cronobacter sakazakii		CGMCC	16 (8.47)
CGMCC 1.1869	Shigella dysenteriae		CGMCC	16 (8.47)
CGMCC 1.10612	Pseudomonas aeruginosa		CGMCC	128 (67.79)
T17	E. coli	ESBL	Clinical isolate	16 (8.47)
T18	E. coli	ESBL	Clinical isolate	8 (4.24)
15A9	E. coli	Carbapenem resistant	Clinical isolate	16 (8.47)
14E-3	E. coli	Carbapenem resistant	Clinical isolate	16 (8.47)
CEC20126	E. coli	Carbapenem resistant	Clinical isolate	32 (16.95)
ATCC 43300	S. aureus	MRSA	ATCC	16 (8.47)
D2-5F2	S. aureus	MRSA	Clinical isolate	32 (16.95)
DN-65	S. aureus	MRSA	Clinical isolate	32 (16.95)
B04070	S. aureus	MRSA	Kangtai Biotechnology Co., Ltd., Wenzhou, China	16 (8.47)
ATCC 51299	E. faecalis	Vancomycin resistant	ATCC	64 (33.90)
CKP1902	Klebsiella pneumoniae	Carbapenem resistant	Clinical isolate	64 (33.90)
CAB21	Acinetobacter baumannii	Carbapenem resistant	Clinical isolate	32 (16.95)

In other discovery processes for AMPs in Eisenia andrei (Bouché, 1972), Bodó et al. found Lumbr and LuRP, which are related to lumbricin, though analyzing mRNA (22). In comparison, a homologue is not needed in the AMP prediction method applied in this study. Taken alongside the structure-function research, the AMP prediction method could help to discover more novel AMPs.

### EWAMP-R tends to insert into the Gram-negative membrane and bind with the Gram-positive membrane surface.

The predicted structure of EWAMP-R is shown in Fig. 1a. The oxygen atom in the carbonyl of Ser5 forms hydrogen bonds with hydrogens in the amide of Gly7 and Trp8. The bonds lead to a β-turn formed in EWAMP-R with Ser5, Gly6, Gly7, and Trp8. The β-turn can add to increased exposure of Trp8 and may enhance the antibacterial activity. A simulation was conducted to show the interaction between EWAMP-R and the membrane structure.

**FIG 2:**
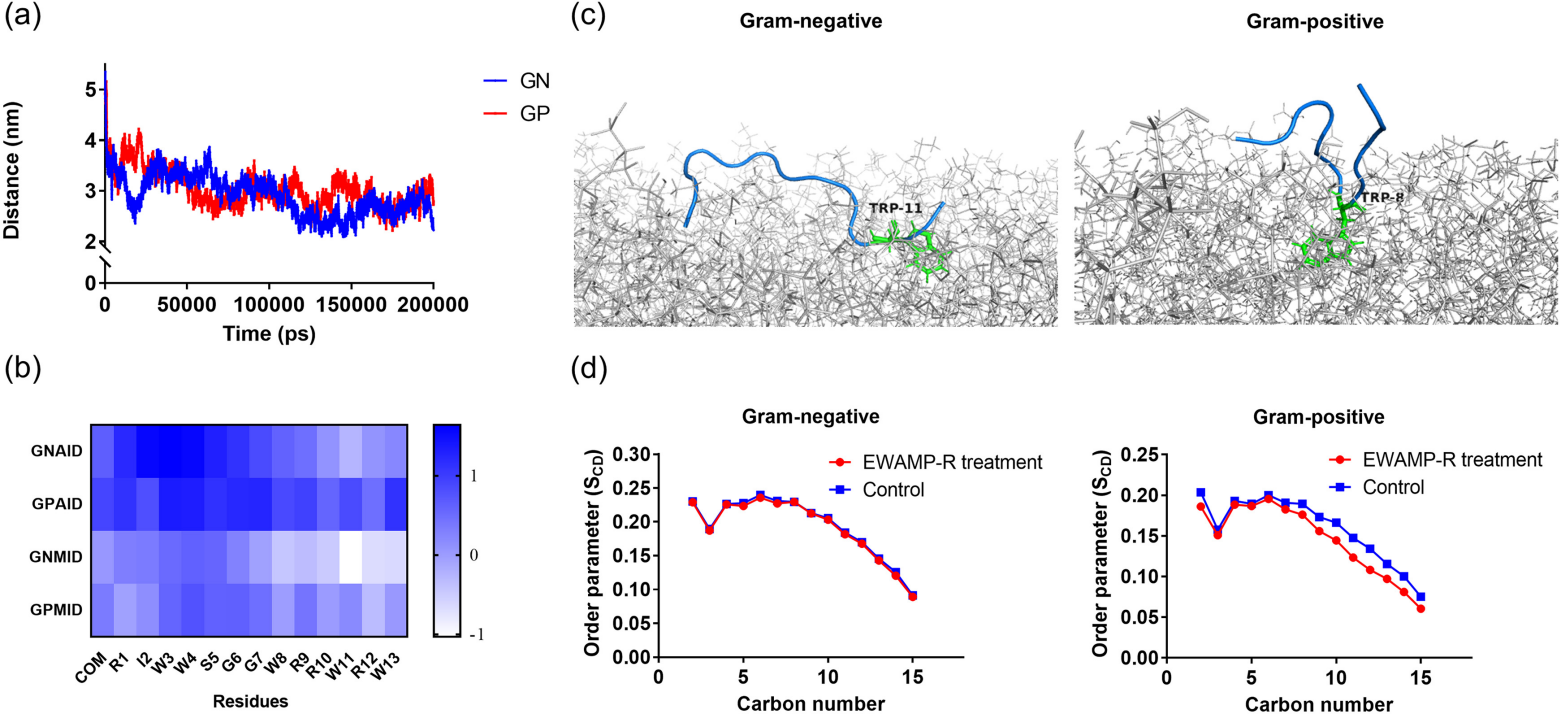
Interaction of EWAMP-R and bacterial membrane in a molecular simulation system. (a) Distance between the center of mass (COM) of EWAMP-R and the COM of bacterial membrane. (b) Insertion depth map of EWAMP-R. The insertion depth = (The distance between the COM of EWAMP-R and the COM of the membrane) − (The distance between the COM of phosphates in the top leaflet and the COM of the membrane). GNAID/GPAID represents the average insertion depth on Gram-negative/-positive bacterial membranes during 150 to 200 ns, and GNMID/GPMID represents the maximum insertion depth on Gram-negative/-positive bacterial membranes during 200 ns. A positive value shows that the residue does not insert into the membrane, and a negative value shows the opposite. (c) Relative positions of EWAMP-R in Gram-negative/-positive systems at 200 ns. (d) S_CD_ of Gram-negative/-positive systems.

During the simulation, EWAMP-R approached the bacterial membrane rapidly, and then limited fluctuations in the distance showed that it was bound to the phospholipids (Fig. 2a). Interestingly, the EWAMP-R molecule was slightly closer to the center of mass (COM) of the Gram-negative membrane than to that of the Gram-positive membrane (Fig. 2b), ultimately regardless of the fact that the Gram-positive membrane was thinner than the Gram-negative membrane (23). This suggests that EWAMP-R could insert deeper into the Gram-negative membrane model, differing from some typical cationic AMPs, like human β-defensin-3 (hBD3) (23), even though there is a greater negative charge in the Gram-positive membrane model. The difference between EWAMP-R and hBD3 suggests that there might be a different interaction and that additional forces besides the electrical charge play an important role in the interaction between EWAMP-R and the membrane.

The distances between the residues and the phosphates of the top phospholipids, especially residues R10, W11, and R12, also showed that EWAMP-R could insert into the Gram-negative bacterial membrane. However, it seems that EWAMP-R would tend to be located at the membrane surface in the Gram-positive membrane model. The relative position at 200 ns also showed the same results. Most residues could get into the Gram-negative membrane, and Trp11 inserted the deepest, while some residues were not intercalated with the Gram-positive membrane, except Trp8, which may result from the β-turn exposing Trp8. Meanwhile, the average deuterium order parameter (S_CD_) values representing the order parameter per atom for carbon tails in the Gram-negative membrane system had little difference between the EWAMP-R group and the control group (Fig. 2d). However, in the Gram-positive membrane group, the S_CD_ of C-7 to C-15 was smaller in the EWAMP-R group, showing that the order of acyl chains was disturbed (Fig. 2d). Although there was no obvious change in membrane thickness (Table S6), the Gram-positive membrane may have been influenced by having more monomers.

**FIG 3:**
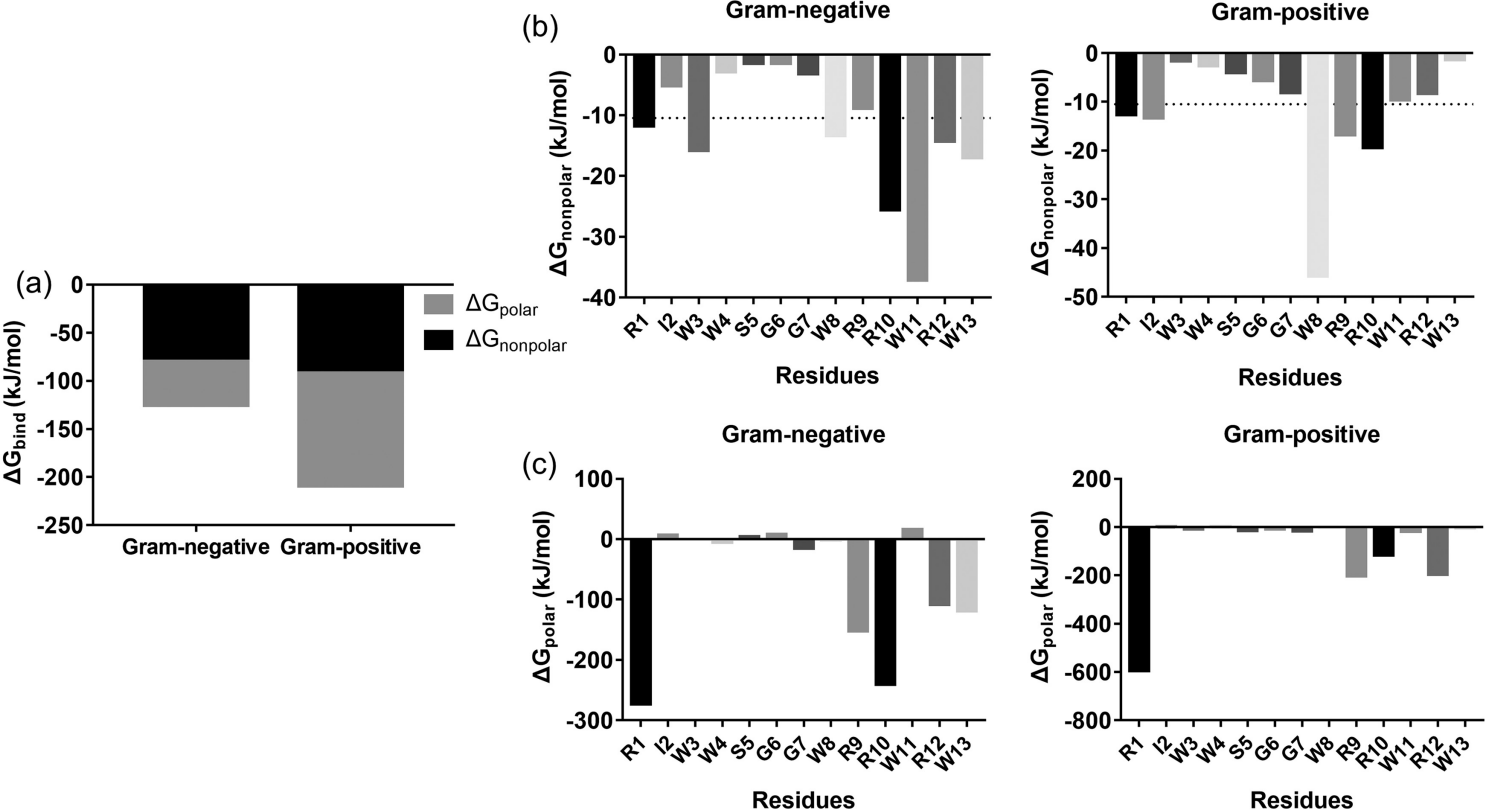
Relative binding free energy in EWAMP-R and bacterial membrane system. (a) Relative binding free energy of Gram-negative/-positive systems. (b) Nonpolar binding energy contributions of EWAMP-R residues to Gram-negative/-positive systems. The residues with significant contributions (<−10.465 kJ/mol, or −2.5 kcal/mol) are shown. (c) Polar binding energy contributions of EWAMP-R residues of Gram-negative/-positive systems.

According to the values for root mean square deviation (RMSD) and root mean square fluctuation (RMSF) (Fig. S3), the Gram-positive membrane system reached equilibrium state rapidly, whereas in the Gram-negative membrane system, it was not equilibrated until 150 ns. During 150 to 200 ns, EWAMP-R formed a more stable binding with the Gram-positive membrane, since it had a smaller RMSF than the Gram-negative membrane system. The relative binding free energy (Δ*G*_bind_) also exhibited the same result, with the Δ*G*_bind_ value for the Gram-positive membrane system being almost twice as large as that of the Gram-negative membrane system (Fig. 3a). In particular, the electrostatic contribution (ΔELE) in the polar interaction contribution (Δ*G*_polar_) showed a significant difference (ΔELE_Gram-negative_ = −49.48 kJ/mol and ΔELE_Gram-positive_ = −120.77 kJ/mol), since the Gram-negative membrane has a greater negative charge. The large difference in ΔELE results and the nonpolar interaction contribution (Δ*G*_nonpolar_) being stronger in the Gram-negative membrane system may explain why it is harder for EWAMP-R to enter the hydrophobic area in the Gram-positive membrane than in the Gram-negative membrane. From the free energy decomposition of EWAMP-R residues, many residues showed effective nonpolar interactions (Δ*G*_nonpolar_ < −10.47 kJ/mol) (Fig. 3c and 3d). Comparing the Gram-negative and Gram-positive membrane systems, the contribution to the free energy of residues was not the same. Trp11 played an important role in nonpolar binding energy in the Gram-negative membrane, while Trp8 had the lowest Δ*G*_nonpolar_ in the Gram-positive membrane system, as shown by the results in Fig. 2c. For polar interactions, Arg residues exhibited significant contributions, especially Arg1. But Arg1 provided more remarkable interactions in the Gram-positive membrane system than in the Gram-negative membrane system. Therefore, it can be conjectured that in the binding process, nonpolar effects play a major role in the Gram-negative membrane system, while polar effects contribute a predominant effect in the Gram-positive membrane system.

### EWAMP-R triggers bacterial cell membrane damage.

**FIG 4:**
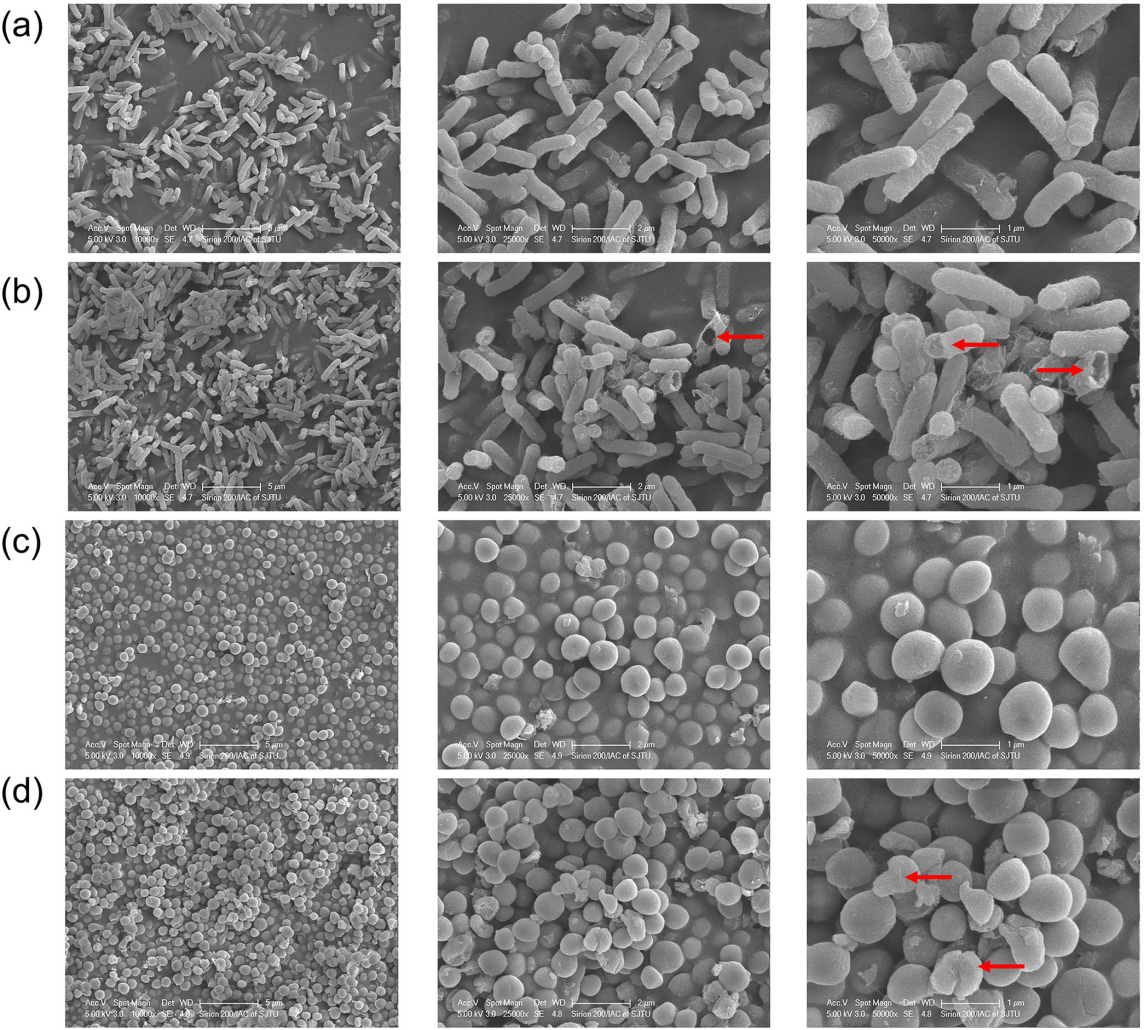
Morphological studies of E. coli and S. aureus before and after EWAMP-R treatment. Bacteria were observed under SEM. (a and c) Control groups of E. coli ATCC 25922 (a) and S. aureus ATCC 29213 (c). (b and d) E. coli ATCC 25922 (b) and S. aureus ATCC 29213 (d) incubated with 80 μg/mL EWAMP-R for 1 h. Red arrows show damaged bacterial cells.

Bacterial cell morphology was observed after EWAMP-R treatment. As shown by scanning electron microscopy (SEM), remarkable membrane damage resembling holes and fractures (Fig. 4b) was observed in E. coli cells and S. aureus cells showed irregular malformation and leakage of cell contents (Fig. 4d) when compared with the typical smooth surface morphology of untreated E. coli and S. aureus bacteria (Fig. 4a and 4c). The observations suggest that EWAMP-R can damage the bacterial membrane.

**FIG 5:**
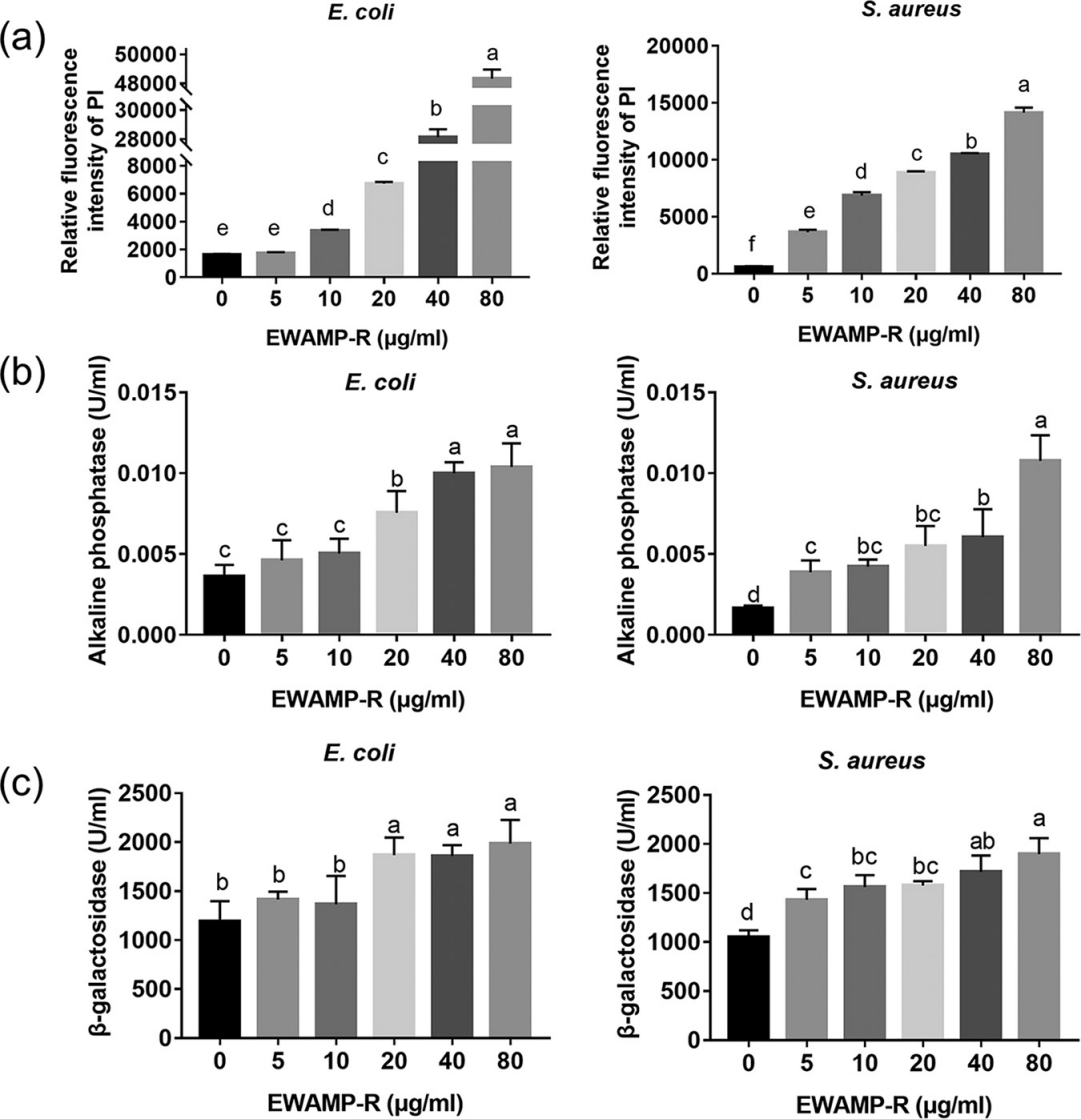
Damage to bacterial cell membrane after treatment with EWAMP-R. (a) Relative fluorescence intensities of propidium iodide (PI), showing the membrane permeability (*n* = 3). (b) Extracellular alkaline phosphatase activities (*n* = 3). (c) Extracellular β-galactosidase activities (*n* = 3). Different letters indicate significant differences determined by ANOVA (*P < *0.05).

Additionally, in order to exhibit the effect of EWAMP-R on the bacterial membrane, membrane damage and cell content leakage were measured. In the observations of bacterial membrane integrity, EWAMP-R dose dependently caused elevation of the fluorescence intensity of propidium iodide (PI) in both E. coli and S. aureus (Fig. 5). Extracellular alkaline phosphatase (ALP) and β-galactosidase activities showed the same trends, such that cell content leakage had a significant positive correlation with EWAMP-R concentrations. In the high-concentration groups, it was consistent with the morphology results; namely, membrane damage was found in both bacteria. However, in the low-concentration groups, membrane damage was not obvious in E. coli.

Combining the wet-laboratory experiments and molecular simulation results, the bacterial membrane may be one of the targets of EWAMP-R, but the peptide may have different interactions with membranes of Gram-negative and Gram-positive bacteria, nevertheless resulting in similar membrane damage in the high-concentration EWAMP-R treatment groups. In the Gram-negative membrane system, EWAMP-R can insert into the membrane due to the stronger nonpolar contribution (Fig. 3a). Weaker binding free energy also can result in relatively unstable binding, so that EWAMP-R can get through the membrane in addition to staying on the surface. The membrane integrity tests also confirm that in the low-concentration groups, EWAMP-R cannot damage the membrane of E. coli, but EWAMP-R has better antibacterial activity against E. coli than S. aureus, indicating that EWAMP-R enters the E. coli cell and affects the cell contents. Therefore, two interaction models between EWAMP-R and the bacterial membrane can be proposed in the low-concentration groups. For Gram-negative bacteria, such as E. coli, EWAMP-R would insert deeply into the membrane and some EWAMP-R molecules might enter into the bacterial cell over time. For Gram-positive bacteria, such as S. aureus, most EWAMP-R molecules would bind to the Gram-positive membrane surface, and this might cause increased lateral pressure near the interface, leading to disruption of the lipid acyl chain (24), and the bacteria could undergo necrosis, causing the leakage of cell contents. Meanwhile, when a high concentration is reached, the bacterial membranes are damaged similarly.

From our results and previous research (20, 25, 26), Trp is a suitable amino acid to facilitate AMPs inserting into the membrane and Arg can contribute to the process of AMP insertion. However, due to the negative charges in the bacterial membrane, especially for the Gram-positive bacteria, a unilateral increase in Arg residue quantity may not be helpful for a peptide to get into the bacterial cell because of the strong polar interaction. Hence, when designing a peptide targeted on the bacterial membrane, the cationic residues may contribute to the binding process, whereas when designing bacterial cell-penetrating peptides, the number of cationic residues should be limited.

### EWAMP-R induces secondary responses.

Considering the different interactions that Gram-negative and Gram-positive bacteria exhibited with the EWAMP-R monomer in the low-concentration groups, if the strength of the interaction with the membrane had a positive correlation with EWAMP-R’s antibacterial activities, the antibacterial effect of EWAMP-R would be greater on Gram-positive bacteria than on Gram-negative bacteria, which is inconsistent with the MIC results. Besides, the evidence shown for membrane integrity also suggests that the bacterial membrane damage in E. coli was limited in low EWAMP-R concentrations. Hence, intracellular secondary responses of bacteria may also be induced and lead to bacterial cell death triggered by a primary response to membrane damage.

**FIG 6:**
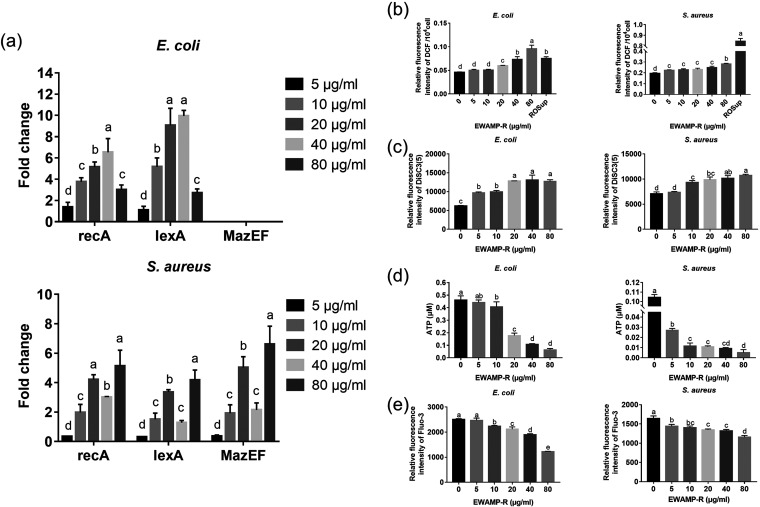
Secondary responses induced by EWAMP-R. (a) Relative gene expression levels of apoptosis-like cell death (ALD) and the MazEF pathway (*n* = 3). Keys show the concentrations of EWAMP-R in culture medium. (b) Relative fluorescence intensities of DCF (dichlorofluorescein), representing the contents of ROS (*n* = 3). (c) Relative fluorescence intensities of DiSC_3_(5) (3,3′-dipropylthiadicarbocyanine iodide), showing changes in membrane potential (*n* = 3). (d) Intracellular ATP contents (*n* = 3). (e) Relative fluorescence intensities of fluo-3 AM, representing the contents of intracellular Ca^2+^ (*n* = 3). Different letters indicate significant differences, determined by ANOVA (*P < *0.05).

Interestingly, after treatment with EWAMP-R, apoptosis-like cell death (ALD) and MazEF pathways were both activated in S. aureus, while only the ALD pathway was stimulated in E. coli (Fig. 6a). For E. coli, the expression levels of *recA* and *lexA* genes were upregulated in all EWAMP-R treatment groups, while the expression of *mazEF* was almost undetectable. In contrast, the expression levels of *recA*, *lexA*, and *mazEF* in S. aureus were downregulated under low-concentration (5 μg/mL) conditions. In the higher-concentration groups, in addition to *recA* and *lexA*, the expression of *mazEF* was also upregulated, implying that the MazEF pathway was also stimulated in S. aureus.

With increasing EWAMP-R concentrations, the content of reactive oxygen species (ROS) increased, and at 80 μg/mL, it was significantly higher than in the Rosup-treated positive control in E. coli (Fig. 6b). Rosup is a 50 μg/mL compound mixture provided with DCFH-DA (Beyotime), which could significantly increase ROS in cells. Bacterial membrane depolarization was found as another related secondary response in the ALD pathway (27). The fluorescence intensity of DiSC_3_(5) (3,3′-dipropylthiadicarbocyanine iodide) increased, revealing that the membrane potential decreased. In the 5-μg/mL concentration groups, the membrane potential was influenced in E. coli but not in S. aureus (Fig. 6c). In the high-concentration groups, due to the membrane damage, the membrane potential in both bacteria was significantly decreased. The intracellular ATP and Ca^2+^ contents also exhibited the same trend in that they were negatively correlated with the EWAMP-R concentration. The cell membrane is the energy metabolism site for bacteria, and the energy in the membrane potential can drive a membrane-bound ATP synthase to phosphorylate ADP to ATP (28); thus, membrane depolarization from EWAMP-R treatment can result in disturbed ATP production and influence energy metabolism. Meanwhile, Ca^2+^ homeostasis is maintained in bacteria by (i) primary ATP-dependent Ca^2+^ pumps and secondary Ca^2+^ transporters to remove Ca^2+^ and (ii) specific proteins or phospholipids in the cell membrane and wall to transport Ca^2+^ into the cytoplasm (29). However, the intracellular Ca^2+^ content decreased with the decline of ATP content, suggesting that the absorption system was damaged, which may have resulted from membrane damage and lipid peroxidation from increased ROS. Disrupted Ca^2+^ homeostasis would disturb a variety of bacterial processes.

Based on these results, it can be conjectured that after EWAMP-R treatment, the ALD pathway was stimulated, leading to the decrease in membrane potential and increase of ROS content, inducing widespread bacterial metabolism, and finally resulting in bacterial death. The different results for E. coli and S. aureus may explain the stronger antibacterial activity of EWAMP-R against E. coli than against S. aureus. MazF can cleave *recA* mRNA and inhibit the influence of the ALD pathway in bacterial cells, including membrane depolarization and DNA fragmentation (27, 30). Hence, through the activation of the MazEF pathway, S. aureus could inhibit the ALD pathway and slow the ROS increase. This is consistent with previous research showing that upregulation of *mazF* correlates with methicillin resistance in S. aureus (31) and that when the *mazF* gene is disrupted, S. aureus is sensitive to antibiotics *in vivo* (32). Therefore, low expression of *mazF* is key in regard to the stronger antibacterial activity against E. coli than against S. aureus.

### EWAMP-R kills E. coli and S. aureus through different mechanisms.

**FIG 7:**
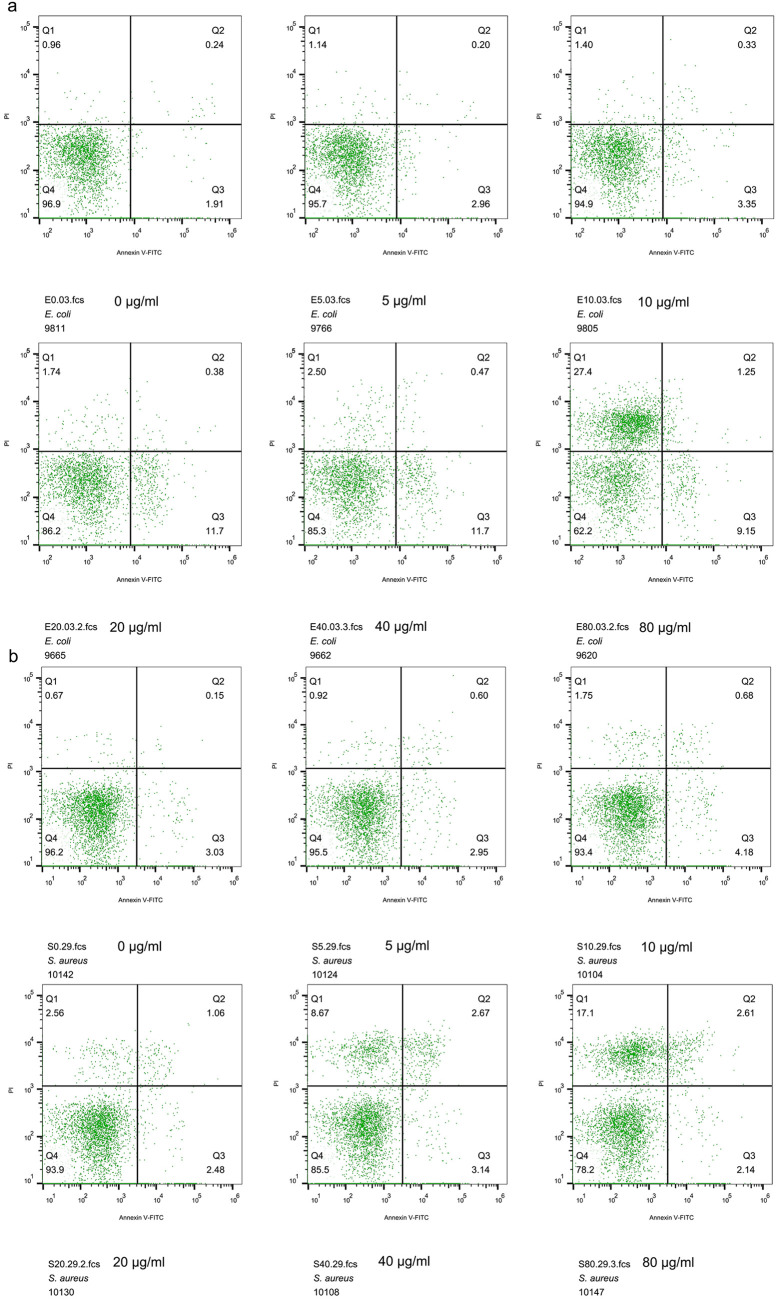
Programed cell death and necrosis rates after treatment with various concentrations of EWAMP-R. The cells were dyed with PI and annexin V-FITC and then analyzed using the CytoFlex flow cytometer. (a) E. coli ATCC 25922. (b) S. aureus ATCC 29213. The labels show the concentrations of EWAMP-R in the culture medium.

As shown by the flow cytometry results in Fig. 7, without EWAMP-R stress, most E. coli and S. aureus cells (>95%) were not fluorescently labeled. In the low-EWAMP-R-concentration groups, a proportion of E. coli cells were labeled with annexin V-fluorescein isothiocyanate (FITC) instead of PI, indicating that apoptosis-like cell death occurred but the bacterial membrane may not have been particularly damaged. Only in the high-concentration groups was there an obvious increase in the number of E. coli cells labeled with PI. In contrast, only a few S. aureus cells (<6%) combined with annexin V-FITC in all groups, while the cell number labeled with PI was positively related to EWAMP-R concentrations. Taking all the results into consideration, it is likely that EWAMP-R may have different antibacterial mechanisms of action on E. coli and S. aureus, in that it kills E. coli mainly through its secondary response, while it kills S. aureus mainly through membrane damage.

In conclusion, to the best of our knowledge, this is the first time a novel AMP has been identified using earthworm genome data. The method we have employed here is rapid, simple, and stable for identifying and optimizing potential novel AMPs when compared to traditional methods of extraction-separation using earthworm body homogenate. The identified AMP, EWAMP-R, showed good antibacterial activity against E. coli and S. aureus, including antibiotic-resistant strains. Its antibacterial activity suggests potential for development into an antibacterial agent, which is confirmed by its action on clinical isolates. Additionally, the different mechanisms employed by EWAMP-R to kill E. coli and S. aureus can deepen the understanding of the quantitative structure-activity relationship of AMPs and will provide basic information for highly specific AMP design. The strong survival ability of earthworms in a complex environment along with the rich biodiversity of earthworm species are indicative of potentially a large library of undiscovered antimicrobial peptides. Moreover, with the completion of genome sequencing in other earthworms, this source of novel antimicrobial peptides will increase and become an extensive resource for the discovery of new antimicrobial agents to combat bacterial resistance to antibiotics.

## MATERIALS AND METHODS

### Bacteria and cell strains.

All bacterial strains were donated by Xianming Shi (Shanghai Jiao Tong University, China), Tao Li and Yingchun Liu (Shanghai Veterinary Research Institute, Chinese Academy of Agriculture Sciences), and Chaoxi Chen (Southwest Minzu University, China). Human normal liver LO2 cells were donated by Xinlin Wei (Shanghai Jiao Tong University). The monoclonal bacteria were cultured in Mueller-Hinton agar medium (Hopebio, Qingdao, China) at 37°C. The cells were cultured in RPMI 1640 medium (Solarbio, Beijing, China) with 10% fetal bovine serum (FBS; Sangon, Shanghai, China) under laboratory conditions of 37°C and 5% CO_2_.

### Discovery and sequence optimization of earthworm antimicrobial peptides.

The open reading frames of the total genome of Eisenia andrei (Bouché, 1972) GWHACBE00000000 (19) were read and translated into amino acid sequences by using ORFfinder (NCBI; https://ftp.ncbi.nlm.nih.gov/genomes/TOOLS/ORFfinder/). The potential AMPs were predicted by CAMP_R3_ (18). All selected peptides were solid state synthesized by GL Biochem Ltd. (Shanghai, China) (purity of >95%).

### Measurement of antibacterial activity.

The broth microdilution method was used to determine the MIC value, following CLSI document M07-A9 (33), in Mueller-Hinton broth medium (Hopebio). Bacteria in the logarithmic phase were diluted to around 10^6^/mL, and different concentrations of the AMP were added. The cultures were gradient diluted and then plated onto tryptic soy agar medium (Hopebio) at 0, 30, 60, 90, and 120 min posttreatment. After plates had been cultured for 24 h at 37°C, the kinetics of antimicrobial activity were visualized through plotting the CFU counts.

### Sensitivity to temperature and pH.

The AMP was treated for 1 h with a water bath heated at 50, 60, 70, 80, 90, or 100°C to evaluate temperature sensitivity, while the pH was adjusted to 2, 4, 6, 8, 10, and 12 and then back to 7.4 after 1 h to evaluate pH sensitivity. The changes in antibacterial activity were determined using the MIC method, as follows: antibacterial rate = [(OD_600 positive control_ − OD_600 sample_)/(OD_600 positive control_ − OD_600 negative control_)] × 100, where OD_600_ is the optical density at 600 nm.

### Toxicity evaluation.

Sterile defibrinated sheep blood (Titan, Shanghai, China) was kept at 4°C and was used within 3 days after being received. Hemolysis assessment was conducted following the method of Liu et al. (34). The hemolysis rate was calculated using the following formula: hemolysis rate = [(*A*_576 sample_ − *A*_576 blank_)/(*A*_576 0.2% Triton X-100_ − *A*_576 blank_)] × 100.

The cytotoxicity of EWAMP-R was also evaluated using the WST-1 cell proliferation and cytotoxicity assay kit (Beyotime, Shanghai, China) with the following formula: cell survival rate = [(Δ*A*_sample_ − Δ*A*_blank control_)/(Δ*A*_negative control_ − Δ*A*_blank control_)] × 100, where Δ*A_i_* = *A*_450_ *_i_* − *A*_655_ *_i_*.

### Molecular simulation and binding free energy calculation.

A phospholipid bilayer with 256 phospholipids was built using CHARMM-GUI (35–38). A 1:3 ratio of palmitoyloleoylphosphatidylglycerol (POPG)/palmitoyloleoylphosphatidylethanolamine (POPE) was simulated to model the cell membrane of Gram-positive bacteria, and a 3:1 ratio for the Gram-negative membrane. The structure of the AMP was predicted by C-I-TASSER (39), and the distance to the center of mass (COM) of the cell membrane was set to 50 Å in the system. GROMACS 2020 (40) with the CHARMM36m force field (41) was used to perform and analyze the simulations. Simulation of the interaction between the AMP and the bacterial membrane was conducted following the method of Lee et al. (23). Binding free energy (Δ*G*_bind_) was calculated using the molecular mechanics Poisson-Boltzmann surface area (MM-PBSA) method and the decomposition scheme in gmx_MMPBSA 1.5.2 (42). The relative binding free energy was used, and the entropy contribution of the peptide was neglected (23). One hundred frames were extracted from a 180- to 200-ns simulation trajectory for Δ*G*_bind_ calculation. The computations in this paper were run on the π 2.0 cluster supported by the Center for High Performance Computing at Shanghai Jiao Tong University.

### Cell membrane integrity assessment.

Overnight bacteria were washed, and the AMP at different concentrations or an equal volume of phosphate-buffered saline (PBS) added. Then, the mixtures were cultured for 1 h at 37°C and centrifuged (4°C at 8,000 × *g* for 5 min). After centrifugation, the sediments were added to 5 μM PI (Sangon) and incubated at 37°C for 30 min. After washing 3 times, the fluorescence intensity was determined using the M1000 multimode reader (Tecan, Hombrechtikon, Switzerland). The supernatant was taken to measure the extracellular ALP and β-galactosidase activity using an alkaline phosphatase assay kit (Beyotime) and a β-galactosidase detection kit (Solarbio).

For morphological observation, the samples were added to 2.5% glutaraldehyde and kept at 4°C for at least 24 h. Then, they were washed 4 times in 0.1 M PBS (4°C for 10 min, followed by centrifugation) and fixed for 1.5 h. Then, they were washed twice in 0.1 M PB and twice in ultrapure water. The samples were dehydrated for 15 min with 50%, 70%, and 90% ethanol successively. Next, the samples were immersed in 100% ethanol for 20 min in triplicate, dried by using a CO_2_ critical point dryer (Leica, Wetzlar, Germany), coated with gold using the Q150T ES plus vacuum coating machine (Quorum, East Sussex, UK), and then observed under SEM (Sirion 200; FEI, USA).

### Programmed cell death studies.

Total RNA of E. coli samples was extracted using the TRIzol kit (Innochem, Beijing, China) and that of S. aureus samples was extracted using the SPARK easy improved bacteria RNA kit (Spark Jade, Shandong, China). They were immediately treated with the PrimeScript reverse transcription (RT) reagent kit with gDNA eraser (TaKaRa, Japan). The mRNA transcriptional expression of *lexA*, *recA*, and *mazEF* was detected by using the CFX Connect real-time system (Bio-Rad Laboratories, USA) and quantified following the cycle threshold (2^−ΔΔ^*^CT^*) method (43). The designed primers for *lexA*, *recA*, and *mazEF* and their internal reference 16S rRNA can be seen in Table S7.

The overnight bacteria were loaded with 0.5 μM DiSC_3_(5) (3,3′-dipropylthiadicarbocyanine iodide; Goyoo, Shanghai, China) and cultured with the AMP at different concentrations at 37°C for 1 h to detect membrane potential. To measure Ca^2+^ levels, the AMP-treated bacteria were added to 5 μM fluo-3 AM (Goyoo) and incubated at 37°C for 30 min. The ATP contents were measured using the Enhanced ATP assay kit (Beyotime). For reactive oxygen species (ROS), the bacteria in the logarithmic phase were adjusted to 10^8^ CFU/mL and 10 μM DCFH-DA (dichlorodihydrofluorescein diacetate; Beyotime) was added. After incubation for 20 min, AMP and Rosup were added and the bacterial fluid was cultured at 37°C for 1 h. All fluorescence intensities and chemiluminescence intensities were determined using the M1000 multimode reader.

### Flow cytometry.

Overnight bacteria with different concentrations of AMP added were cultured for 1 h at 37°C. After dilution to 10^7^ CFU/mL, the bacteria were dyed using the annexin V-FITC apoptosis detection kit (Beyotime) and then detected and analyzed using the CytoFlex flow cytometry instrument (Beckman Coulter, Indianapolis IN, USA) and FlowJo 10.8.1 (BD Life Sciences, USA).

### Statistical analysis.

Triplicates were set in this study, and all the results are expressed as mean values ± standard errors. Statistical analyses were conducted through SPSS 22.0. The different comparisons were tested by Kruskal-Wallis nonparametric one-way analysis of variance (ANOVA) followed by Dunn’s *post hoc* multiple-comparison test. Pictures were drawn by using GraphPad Prism 7. Different letters are used in some bar charts to show significant differences (*P* < 0.05). The results were ranked from high to low level.
