# Supplementary material for: Discovery and Mechanism of Action of a Novel Antimicrobial Peptide from an Earthworm
Source: Microbiol Spectr. 2023 Jan 5;11(1):e03206-22. doi: 10.1128/spectrum.03206-22 (PMC9927515; doi:10.1128/spectrum.03206-22)
Supplement: Supplemental file 1 — Supplemental material. Download spectrum.03206-22-s0001.pdf, PDF file, 0.6 MB [file spectrum.03206-22-s0001.pdf]

Discovery and mechanism of action of a novel antimicrobial peptide from earthworm

Yizhao Wu [1, 2], Songge Deng [1, 2], Xiuhong Wang [1, 2], Michelle Thunders [3], Jiangping Qiu [1, 2], Yinsheng Li* [1, 2]

[1] School of Agriculture and Biology, Shanghai Jiao Tong University, Shanghai, 200240, China

[2] Shanghai Yangtze River Delta Eco-environmental Change and Management Observation and Research Station, Ministry of Science and Technology, Shanghai, 200240, China

[3] Department of Pathology and Molecular Medicine, University of Otago, Wellington, 6242, New Zealand

* Yinsheng Li handled correspondence at all stages of refereeing and publication, also post-publication. Tel: +86 021-34206933. E-mail: yinshengli@sjtu.edu.cn.

Competing Interests statement

The authors declare that they have no known competing financial interests or personal relationships that could have appeared to influence the work reported in this paper.

Legends

Table S1 Sequences of 21 synthetic peptides

Table S2 MICs of 5 AMPs

Table S3 Sequences and MICs of derivatives of EWAMP.15

Table S4 Sequences and MICs of Alanine scanning derivatives

Table S5 Sequences and MICs of Arg and Lys derivatives

Table S6 Summary of membrane thickness

Table S7 Primers used in RT-PCR

Fig. S1 MICs of EWAMP.15 12-24 derivatives that were replaced in different sites. In the X-axis, the number and the letter represent the replaced site and the amino acid residue.

Fig. S2 Physical stability and in vitro toxicity of EWAMP-R. (a) Residual antibacterial rates after water heating for 1 h showing the temperature stability. (b) Residual antibacterial rates after acid or alkaline solution treatment showing the pH stability. (c) Hemolytic activity of EWAMP-R. The absorbance was not significantly different in treatment groups and negative controls. (d) Cell survival rate of LO2 after EWAMP-R treatment. All the cell survival rate was above 95%.

Fig. S3 Root-mean-squared deviation (RMSD) and root-mean-squared fluctuation (RMSF) of EWAMP-R in GN and GP systems. (a) RMSD. (b) RMSF in 150 - 200 ns.

Table S1

Name	Number of amino acid residues	Sequence
EWAMP.1	25	MGYCCCCRCSKGGAFCGALASAPCA
EWAMP.2	22	MCCRGCKRPALTATICCCCGAR
EWAMP.3	25	MCSCCIVKLADCHVLCSCKSCLKRG
EWAMP.4	23	MCRRCPCLGRKCQYIYRRCFCIC
EWAMP.5	25	MCVCRRACVCARVCACVRVSACVHA
EWAMP.6	27	MACNRCLRCGCCKRCCLKSCLDWTRYN
EWAMP.7	25	MCALECVRVGARGCARACGCSCNIR
EWAMP.8	24	MCFKRLDAGAKHCVGCRASLCCCK
EWAMP.9	23	MWRCVATRRRCAAVCRNCAAARR
EWAMP.10	23	MCCRCCFWGRRCLGAGIIRQERR
EWAMP.11	27	MCCLPKCYQCLRFTSCIVYSKGCGRKR
EWAMP.12	25	MLSAYNVCQVKYDGGICCKKKGQCS
EWAMP.13	22	MCGLTKGCGWSTCLGCKVKRVC
EWAMP.14	25	MILRCTGCSHTQNCRCAIKASCIAF
EWAMP.15	25	MLRKVGVIGHWKIWWSGGWKRWRWR
EWAMP.16	24	MCLRRCPSRVYACAYCANVLGCKN
EWAMP.17	25	MGLSCSFLPSYSSSIRCIGIGCCKG
EWAMP.18	25	MCCGCSRWGIAIVCSRKNSSCRNSP
EWAMP.19	23	MLCVRGSGRRNCTWGCCIKCAHS
EWAMP.20	27	MAACGLRVRACLRVCACIYQATQAAKI
EWAMP.21	27	MAACGLRVRACVRVCACIYQATQAAKI

Table S2

Name	Sequence	MIC$_{E. coli}$	MIC$_{S. aureus}$
EWAMP.2	MCCRGCKRPALTATICCC	1024 µg/ml	512 µg/ml
	CGAR	441.2 nmol/ml	220.6 nmol/ml
EWAMP.4	MCRRCPCLGRKCQYIYR	1024 µg/ml	512 µg/ml
	RCFCIC	356.1 nmol/ml	178.1 nmol/ml
EWAMP.9	MWRCVATRRRCAAVCR	1024 µg/ml	512 µg/ml
	NCAAARR	381.9 nmol/ml	191.0 nmol/ml
EWAMP.11	MCCLPKCYQCLRFTSCI	256 µg/ml	256 µg/ml
	VYSKGCGRKR	81.32 nmol/ml	81.32 nmol/ml
EWAMP.15	MLRKVGVIGHWKIWWS	128 µg/ml	128 µg/ml
	GGWKRWRWR	39.19 nmol/ml	39.19 nmol/ml

Table S3

Name	Sequence	MIC$_{E. coli}$	MIC$_{S. aureus}$
EWAMP.15 2-15	LRKVGVIGHWKIWW	64 μg/ml<br>35.99 nmol/ml	64 μg/ml<br>35.99 nmol/ml
EWAMP.15 2-14	LRKVGVIGHWKIW	128 μg/ml<br>80.40 nmol/ml	512 μg/ml<br>160.81 nmol/ml
EWAMP.15 11-24	WKIWWSGGWKRWRW	64 μg/ml<br>31.71 nmol/ml	16 μg/ml<br>7.93 nmol/ml
EWAMP.15 11-23	WKIWWSGGWKRWR	32 μg/ml<br>17.47 nmol/ml	32 μg/ml<br>17.47 nmol/ml
EWAMP.15 11-22	WKIWWSGGWKRW	128 μg/ml<br>76.37 nmol/ml	64 μg/ml<br>38.19 nmol/ml
EWAMP.15 12-24	KIWWSGGWKRWRW	16 μg/ml<br>8.73 nmol/ml	16 μg/ml<br>8.73 nmol/ml
EWAMP.15 13-24	IWWSGGWKRWRW	32 μg/ml<br>18.78 nmol/ml	64 μg/ml<br>37.56 nmol/ml
EWAMP.15 12-23	KIWWSGGWKRWR	64 μg/ml<br>38.88 nmol/ml	128 μg/ml<br>77.77 nmol/ml

Table S4

**Name**	**Sequence**	**MIC$_{E. coli}$**	**MIC$_{S. aureus}$**
EWAMP.15 12-24.01A	AIWWSGGWKRWRW	64 μg/ml<br>36.06 nmol/ml	64 μg/ml<br>36.06 nmol/ml
EWAMP.15 12-24.02A	KAWWSGGWKRWRW	128 μg/ml<br>75.51 nmol/ml	128 μg/ml<br>75.51 nmol/ml
EWAMP.15 12-24.03A	KIAWSGGWKRWRW	256 μg/ml<br>149.1 nmol/ml	＞256 μg/ml<br>＞149.1 nmol/ml
EWAMP.15 12-24.04A	KIWASGGWKRWRW	64 μg/ml<br>37.27 nmol/ml	128 μg/ml<br>74.55 nmol/ml
EWAMP.15 12-24.05A	KIWWAGGWKRWRW	64 μg/ml<br>35.24 nmol/ml	64 μg/ml<br>35.24 nmol/ml
EWAMP.15 12-24.06A	KIWWSAGWKRWRW	64 μg/ml<br>34.67 nmol/ml	64 μg/ml<br>34.67 nmol/ml
EWAMP.15 12-24.07A	KIWWSGAWKRWRW	16 μg/ml<br>8.67 nmol/ml	32 μg/ml<br>17.33 nmol/ml
EWAMP.15 12-24.08A	KIWWSGGAKRWRW	128 μg/ml<br>74.55 nmol/ml	256 μg/ml<br>149.1 nmol/ml
EWAMP.15 12-24.09A	KIWWSGGWARWRW	256 μg/ml<br>144.2 nmol/ml	256 μg/ml<br>144.2 nmol/ml
EWAMP.15 12-24.10A	KIWWSGGWKAWRW	256 μg/ml<br>146.5 nmol/ml	＞256 μg/ml<br>＞146.5 nmol/ml
EWAMP.15 12-24.11A	KIWWSGGWKRARW	128 μg/ml<br>74.55 nmol/ml	256 μg/ml<br>149.1 nmol/ml
EWAMP.15 12-24.12A	KIWWSGGWKRWAW	128 μg/ml<br>73.27 nmol/ml	128 μg/ml<br>73.27 nmol/ml
EWAMP.15 12-24.13A	KIWWSGGWKRWRA	＞256 μg/ml<br>＞149.1 nmol/ml	＞256 μg/ml<br>＞149.1 nmol/ml

Table S5

Name	Sequence	$MIC_{\textit{E. coli}}$	$MIC_{\textit{S. aureus}}$
EWAMP.15 12-24	KIWWSGGWKRWRW	16 μg/ml<br>8.73 nmol/ml	16 μg/ml<br>8.73 nmol/ml
EWAMP.15 12-24. 01R	RIWWSGGWKRWRW	32 μg/ml<br>17.20 nmol/ml	64 μg/ml<br>34.40 nmol/ml
EWAMP.15 12-24. 09R	KIWWSGGWRRWRW	32 μg/ml<br>17.20 nmol/ml	64 μg/ml<br>34.40 nmol/ml
EWAMP.15 12-24. 10K	KIWWSGGWKKWRW	128 μg/ml<br>70.95 nmol/ml	128 μg/ml<br>70.95 nmol/ml
EWAMP.15 12-24. 12K	KIWWSGGWKRWKW	32 μg/ml<br>17.74 nmol/ml	64 μg/ml<br>35.47 nmol/ml
EWAMP.15 12-24. 01 09R	RIWWSGGWRRWRW	8 μg/ml<br>4.24 nmol/ml	16 μg/ml<br>8.47 nmol/ml
EWAMP.15 12-24. 10 12K	KIWWSGGWKKWKW	128 μg/ml<br>72.07 nmol/ml	256 μg/ml<br>144.1 nmol/ml

Table S6

System	Membrane thickness (nm)[a]
Gram-negative membrane system	4.05 (1)
EWAMP-R and gram-negative membrane system	4.02
Gram-positive membrane system	3.83 (1)
EWAMP-R and gram-positive membrane system	3.77

a: Membrane thickness was average distance between center of mass of phosphates in the top and bottom leaflets.

Table S7

Name	Sequence
E-recA-F	AGATCCTCTACGGCGAAGGT (2)
E-recA-R	CCTGCTTTCTCGATCAGCTT (2)
E-lexA -F	GACTTGCTGGCAGTGCATAA (2)
E-lexA -R	TCAGGCGCTTAACGGTAACT (2)
E- mazEF-F	CTTCGTTGCTCCTCTTGC (2)
E- mazEF -R	CGTTGGGGAAATTCACCG (2)
S-recA-F	GCCGAAGCATTTGTTAGAAGTGGTG
S-recA-R	CGTGAGTGTCTCCCATTTCTCCTTC
S-lexA-F	GCAGGTGTTCCTATTACCGCAGTAG
S-lexA-R	GCCTCAATCATACTGTCGCCTACG
S-mazEF-F	GGGAGTCAGACCTGTAGTCA
S-mazEF-R	GTGTCGGTATTTTCGCTTTA
16SrRNA -F	TGTAGCGGTGAAATGCGTAGA (2)
16SrRNA-R	CACCTGAGCGTCAGTCTTCGT (2)

Fig. S1

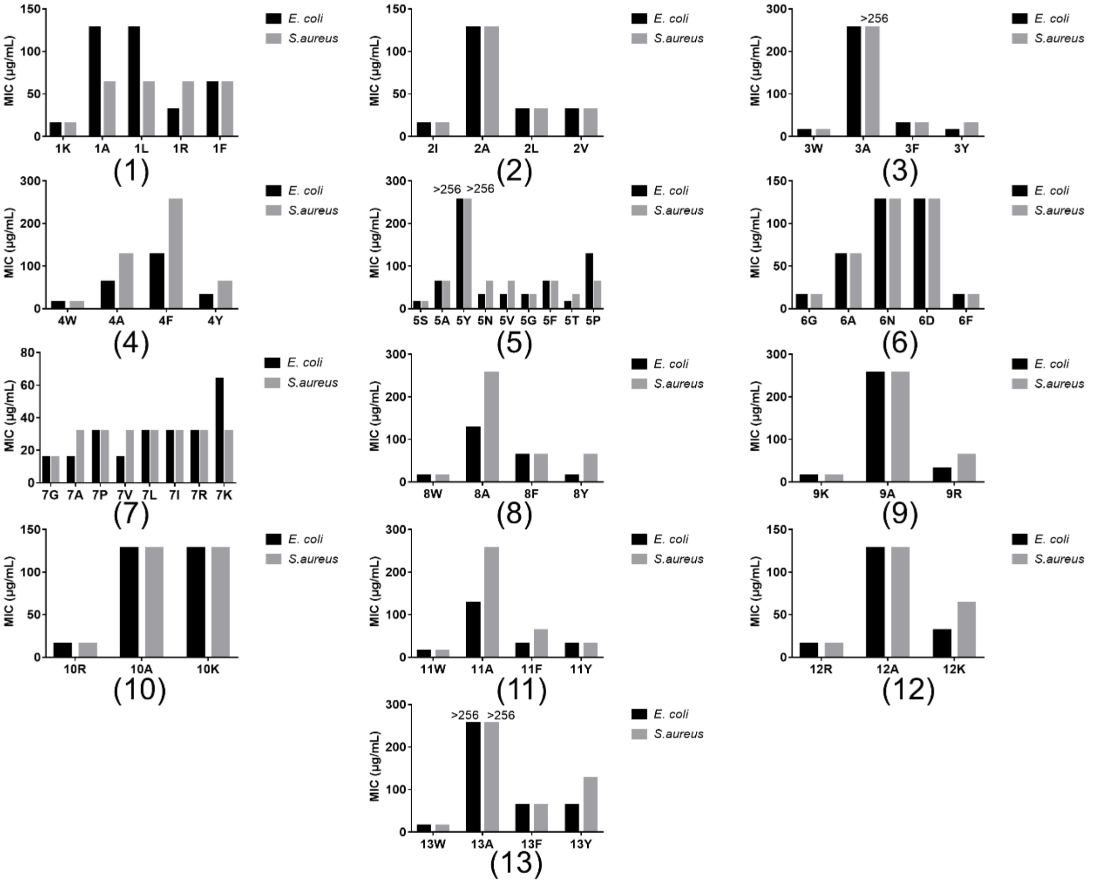
MIC (μg/mL)
E. coli
S.aureus
>256
(1)
(2)
(3)
(4)
(5)
(6)
(7)
(8)
(9)
(10)
(11)
(12)
(13)
1K 1A 1L 1R 1F
2I 2A 2L 2V
3W 3A 3F 3Y
4W 4A 4F 4Y
5S 5A 5Y 5N 5V 5G 5F 5T 5P
6G 6A 6N 6D 6F
7G 7A 7P 7V 7L 7I 7R 7K
8W 8A 8F 8Y
9K 9A 9R
10R 10A 10K
11W 11A 11F 11Y
12R 12A 12K
13W 13A 13F 13Y

Fig. S2

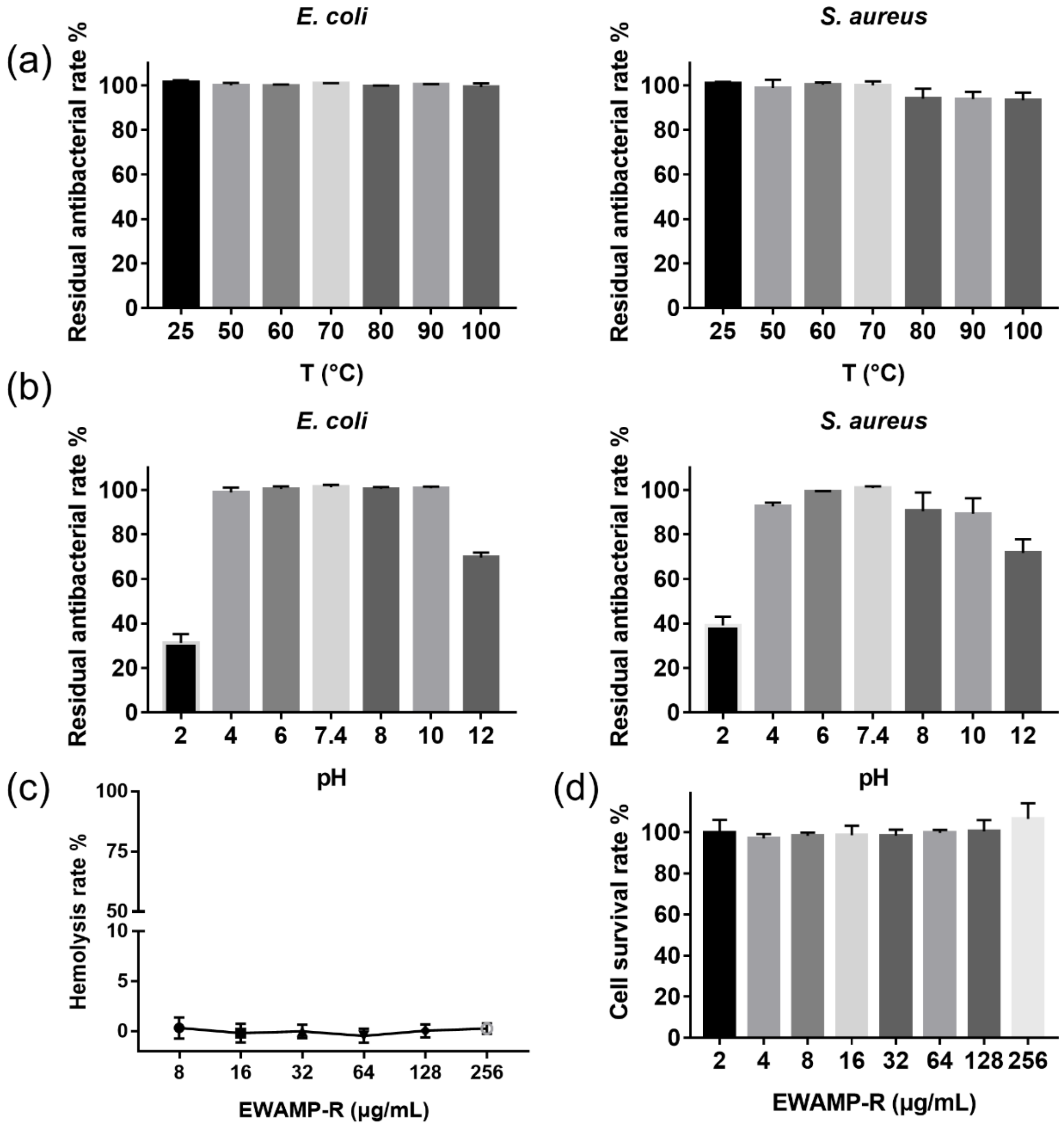
(a)
E. coli
S. aureus
Residual antibacterial rate %
0
20
40
60
80
100
25 50 60 70 80 90 100
T (°C)
(b)
E. coli
S. aureus
Residual antibacterial rate %
2 4 6 7.4 8 10 12
pH
(c)
Hemolysis rate %
0
5
10
50
75
100
8 16 32 64 128 256
EWAMP-R (μg/mL)
(d)
Cell survival rate %
2 4 8 16 32 64 128 256
EWAMP-R (μg/mL)

Fig. S3

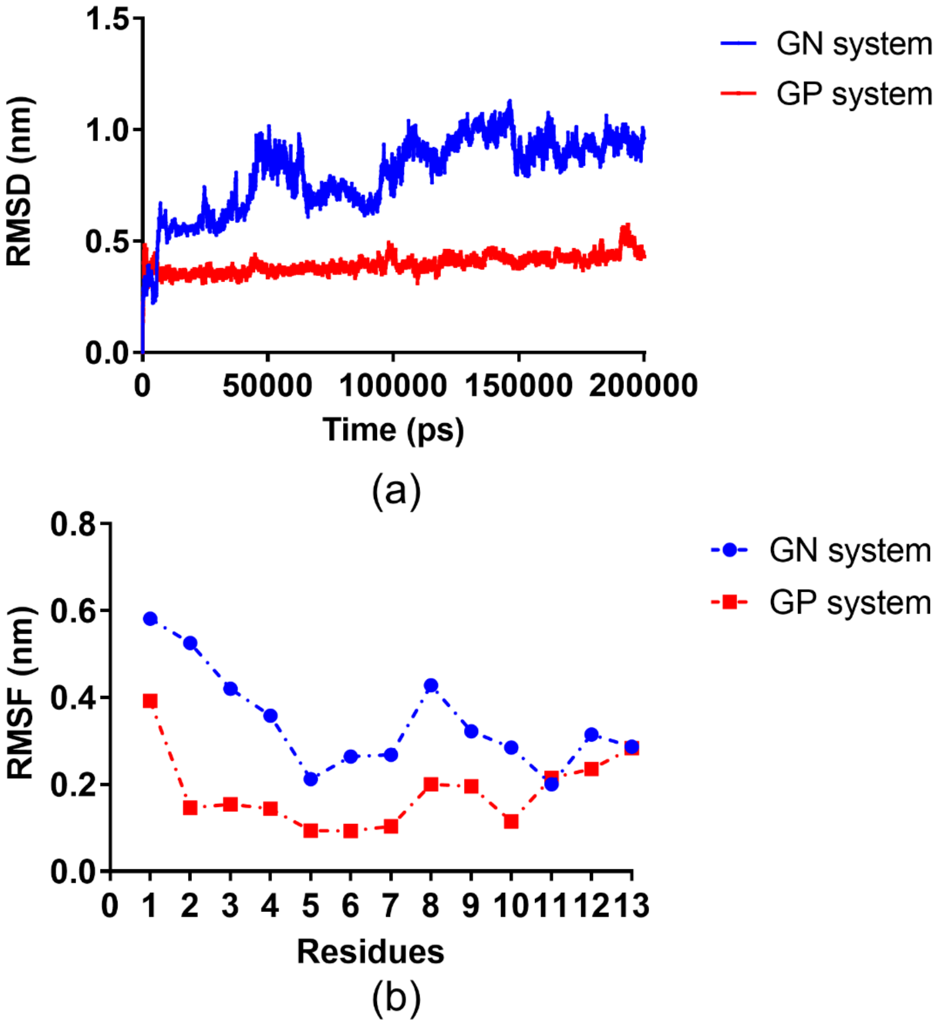
1.5
1.0
0.5
0.0
RMSD (nm)
0
50000
100000
150000
200000
Time (ps)
GN system
GP system
(a)
0.8
0.6
0.4
0.2
0.0
RMSF (nm)
0 1 2 3 4 5 6 7 8 9 10 11 12 13
Residues
GN system
GP system
(b)

References

1. Lee J, Jung SW, Cho AE. 2016. Molecular Insights into the Adsorption Mechanism of Human beta-Defensin-3 on Bacterial Membranes. Langmuir 32:1782-90.

2. Lam SJ, O'Brien-Simpson NM, Pantarat N, Sulistio A, Wong EH, Chen YY, Lenzo JC, Holden JA, Blencowe A, Reynolds EC, Qiao GG. 2016. Combating multidrug-resistant Gram-negative bacteria with structurally nanoengineered antimicrobial peptide polymers. Nature Microbiology 1:16162.
